# The expression of PDGF-BB predicts curative effect in locally advanced esophageal squamous cell carcinoma treated by radiotherapy

**DOI:** 10.18632/aging.102993

**Published:** 2020-04-24

**Authors:** Puchun Er, Dong Qian, Wencheng Zhang, Baozhong Zhang, Hui Wei, Tian Zhang, Xi Chen, Yuwen Wang, Jingjing Zhao, Qi Wang, Qingsong Pang, Ping Wang

**Affiliations:** 1 Department of Radiotherapy, Tianjin Medical University Cancer Institute and Hospital, National Clinical Research Center for Cancer, Key Laboratory of Cancer Prevention and Therapy, Tianjin, Tianjin’s Clinical Research Center for Cancer, Tianjin, China; 2 Department of Radiotherapy, The First Affiliated Hospital of University of Science and Technology of China, Hefei, China

**Keywords:** chemoradiotherapy, esophageal squamous cell carcinoma, serum biomarkers, gene expression, curative effect prediction

## Abstract

Radiotherapy is the major approach and is well tolerated in locally advanced esophageal squamous cell carcinoma (ESCC). And nowadays, no effective biological markers have been identified for predicting the prognosis of patients with ESCC. Platelet-derived growth factor (PDGF) is associated with a poor prognosis of various malignancies. The present study aimed to assess the effect of PDGF-BB on radiotherapeutic responses of ESCC and the underlying mechanisms of its roles in ESCC. Serum from 68 cases that received neoadjuvant or radical radiotherapy was obtained before and during radiotherapy. Gene expression analyses were validated by enzyme linked immunosorbent assay. The prognosis of patients with significantly reduced PDGF-BB was probably better than that of the others found in the progression-free survival and overall survival groups. Depletion of PDGFB significantly suppressed the proliferation, invasion and migration of cancer cells. Inhibiting PDGFB induced cellular apoptosis and promoted the sensitivity to ionizing radiation (IR). Furthermore, IR inhibited PDGF-BB-induced migration by blocking the PI3K/AKT pathway in ESCC cells. We found that the expression of PDGF-BB provided a possible model for predicting ESCC radiotherapy. It can also be used as a prognostic indicator for locally advanced ESCC that was treated by radiotherapy.

## INTRODUCTION

Esophageal cancer (EC) is a highly common and aggressive tumor which is a leading cause of cancer-related deaths worldwide [1]. The estimates of new cases of EC is about 300 thousand worldwide each year. And China has the highest incidence and mortality rate of EC in the world, which accounts for more than half of the global total. Unlike high incidence of esophageal adenocarcinoma in western countries, esophageal squamous cell carcinoma (ESCC) accounts for over 90% of ECs in China. Apart from surgical resection, radiotherapy is a major therapeutic approach of ESCC [2, 3]. However, patients with EC usually have a poor prognosis despite receiving standard chemoradiotherapy. The survival rates at three years post-therapy was approximately only 30-40 percent [4–6].

Human platelet-derived growth factor (PDGF), a potent mitogen for cells of mesenchymal origin, was identified as the serum component responsible for the proliferation of arterial smooth muscle cells [7]. The PDGF family consists of four ligands, PDGF-A, B, C, and D. PDGF-B is involved in the maintenance of microvessels and recruitment of pericytes [8]. PDGF-BB, a homodimer of PDGFB, has been reported to be overexpression in some human tumors and associated with a poor prognosis.

However, there remains a lack of empirical data on the association between PDGF-BB and ESCC. Today, in the era of personalized and precision medicine, the importance of predictive biomarkers and targeted therapy have significantly increased in relevance over recent years. A meta-analysis showed that low expression of COX2, miR-200c, ERCC1 and TS, or high expression of CDC25B and p16, represent potential biomarkers for predicting the response of EC patients following chemoradiotherapy [9]. However, the research suffered from many limitations including a lack of prospective studies and a large sample size. There were significant differences in the general conditions and treatment protocols of the patients included in the meta-analysis. And no mechanism study was involved. High-quality testing analysis of both tumor tissue and serum specimens is worth studying further to make improvements.

Thus, we aimed to identify the function of PDGF-BB as a prognostic biomarker in patients with ESCC who receive radiotherapy. In the present study, we investigated the pivotal role of PDGF-BB in ESCC progression by assessing the expression in serum and tissue specimens. In addition, a series of experiments were carried out using ESCC cell-lines with the aim of exploring the potential effects and mechanism of PDGF-BB in vitro.

## RESULTS

### Significantly reduced of PDGF-BB in serum predicts a better prognosis

Within 36 months of the median time to follow-up, average survival time of all patients was 27.3 months (4.07-47.84 months). The total number of deaths was 42, most of which died of distant metastasis or primary progression.

**Figure 1:**
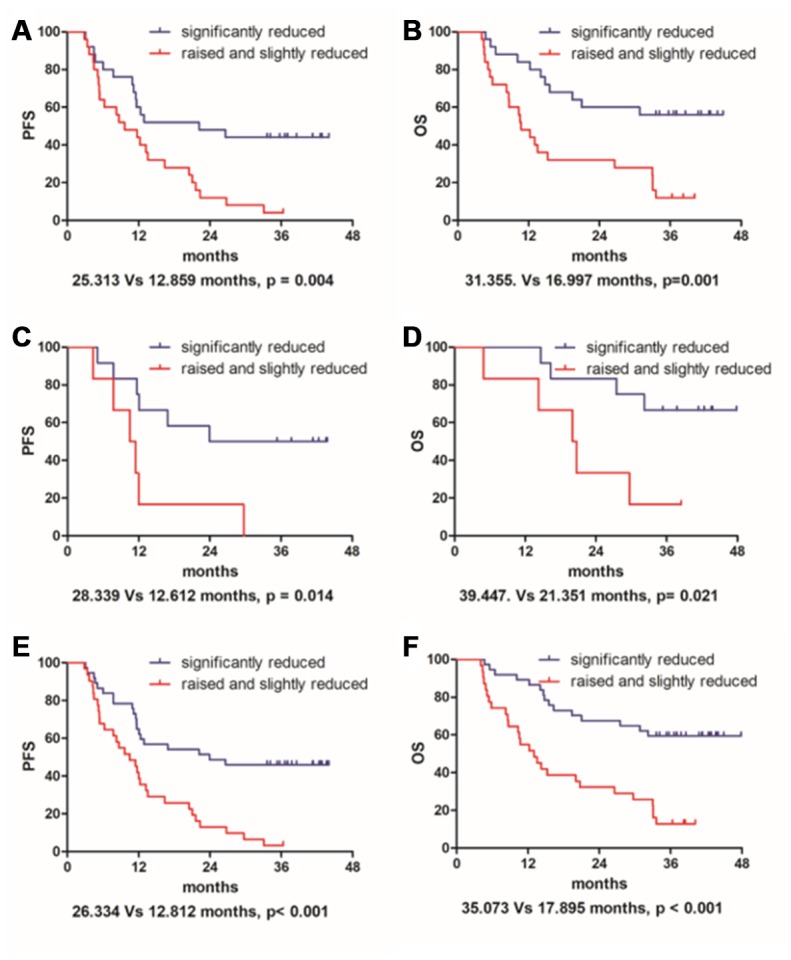
Change rate in serum indicated that patients with significantly reduced PDGF-BB had a much improved prognosis than the raised and slightly reduced group in both progression-free survival (**A**, **C** and **E**) and overall survival (**B**, **D** and **F**) either for the 50 patients that received radical radiotherapy (**A** and **B**) or for the remaining 18 patients that received neoadjuvant radiotherapy and surgery (**C** and **D**). In addition, regarding the entire 68 cases taken together (**E** and **F**), it also has statistical significance.

Regarding the 50 patients that received radical radiotherapy, Kaplan-Meier survival analysis showed that the mean PFS time for patients with significantly reduced levels of PDGF-BB was 25.3 months. In addition, it was only 12.9 months for the group of raised and slightly reduced (25.3 Vs 12.9 months, p = 0.004, shown in Figure 1A). Besides, analysis also showed that the mean OS time for patients with significantly reduced PDGF-BB was 31.4 months, and it was only 17. 0 months for the raised and slightly reduced group (31.4. Vs 17.0 months, p=0.001, shown in Figure 1B). For the remaining 18 patients that received neoadjuvant radiotherapy and surgery, the mean PFS time for patients with significantly reduced was 28.3 months, and it was only 12.6 months for the group of raised and slightly reduced (28.3 Vs 12.6 months, p = 0.014, shown in Figure 1C). The mean OS time for patients with significantly reduced was 39.4 months while it was 21.4 months for the other group (39.4. Vs 21.4 months, p=0.021, shown in Figure 1D).

Furthermore, for the entire 68 cases taken together, statistical significance was also found in PFS (26.33 Vs 12.81 months, p < 0.001, shown in Figure 1E) and OS (35.07. Vs 17.90 months, p< 0.001, shown in Figure 1F). According to the Chi-square test, change rates of PDGF-BB were probably associated with response to radiotherapy (p = 0.042). It was independent of gender, age, tumor location and other clinic variables (p > 0.05). Details are shown in Table 1. Tumor location may significantly affect prognostic factors associated with OS by univariate and multivariate analysis. It showed that OS was more prolonged in the group of patients with an upper EC (p = 0.021 and p = 0.022, shown in Tables 2 and 3). Besides the change rate and response, multivariate analysis also showed concurrent chemotherapy might be a prognostic factor for PFS (p = 0.033, Tab.3). However, there was no significant survival benefit for surgical patients.

**Table 1:** Association of change rate of PGDF-BB with clinicopathological variables.

**Variable**	**n**	**Change rate of PGDF-BB**		**P-value**
		**raised and slightly reduced (n=31)**	**significantly reduced (n=37)**	
**Gender**				0.790
male	60	27	33	
female	8	4	4	
**Age, years**				0.411
<60	31	12	19	
≥60	37	19	18	
**Smoking**				0.280
yes	53	26	27	
no	15	5	10	
**KPS**				0.204
<90	15	9	6	
≥90	53	22	31	
**UGI type**				0.941
fungating type	26	12	14	
other types	42	19	13	
**TNM stage**				0.115
I-II	14	9	5	
III-IV	54	22	32	
**Multifocal lesions**				0.941
yes	9	4	5	
no	59	27	32	
**Tumor location**				0.910
upper	18	8	10	
middle+ lower	50	23	27	
**Surgical resection**				0.223
yes	18	6	12	
no	50	25	25	
**Concurrent chemotherapy**				0.278
yes	62	27	35	
no	6	4	2	
**Response**				0.042*
CR+PR+SD	57	23	34	
PD	11	8	3	

KPS, karnofsky performance scale; UGI, Upper Gastrointestinal Imaging; SD, stable disease; PD, progressive disease; CR, complete response; PR, partial response; *, P < 0.05.

**Table 2:** Prognostic factors for PFS and OS in univariate analysis.

**Prognostic factors (n)**	**Median PFS (months)**	**Median OS (months)**	**P of PFS**	**P of OS**
**Gender**			0.392	0.983
male (60)	19.633	27.652		
female (8)	25.065	26.494		
**Age, years**			0.894	0.759
<60 (31)	19.611	27.683		
≥60 (37)	20.712	26.713		
**Smoking**			0.655	0.792
yes (53)	19.895	27.306		
no (15)	21.701	27.449		
**KPS**			0.463	0.522
<90 (15)	17.415	23.186		
≥90 (53)	21.010	28.471		
**UGI type**			0.740	0.601
fungating type (26)	19.132	26.289		
other types (42)	20.516	26.343		
**TNM stage**			0.722	0.979
I-II (14)	22.419	27.924		
III-IV (54)	19.669	27.367		
**Multifocal lesions**			0.973	0.921
yes (9)	17.719	26.429		
no (59)	20.437	27.740		
**Tumor location**			0.059	0.021*
upper (18)	26.788	36.743		
middle+ lower (50)	17.908	23.569		
**Surgical resection**			0.384	0.130
yes (18)	23.097	33.937		
no (50)	19.242	24.464		
**Concurrent chemotherapy**			0.164	0.084
yes (62)	20.923	28.669		
no (6)	12.507	15.822		
**Response**			<0.001***	<0.001***
CR+PR+SD (57)	23.127	31.374		
PD (11)	5.543	8.627		
**Change rate of PDGF-BB**			<0.001***	<0.001***
SR (37)	26.334	35.073		
RSR (31)	12.812	17.895		

KPS, karnofsky performance scale; UGI, Upper Gastrointestinal Imaging; SD, stable disease; PD, progressive disease; CR, complete response; PR, partial response; RSR, raised and slightly reduced; SR, significantly reduced. *, P < 0.05; ***, P < 0.001.

**Table 3:** Prognostic factors for PFS and OS in multivariate analysis.

**Prognostic factors (n)**	**Hazard ratio of PFS (95% CI)**	**Hazard ratio of OS (95% CI)**	**P of PFS**	**P of OS**
**Tumor location**			0.083	0.022*
upper (18)	1	1		
middle+ lower (50)	2.013 (0.912-4.442)	2.873 (1.161-7.109)		
**Surgical resection**			0.979	0.323
yes (18)	1	1		
no (50)	1.010 (0.492-2.701)	1.501 (0.671-3.356)		
**Concurrent chemotherapy**			0.033*	0.079
yes (62)	1	1		
no (6)	3.130 (1.099-8.918)	2.523 (0.899-7.083)		
**Response**			<0.001***	0.005**
CR+PR+SD (57)	1	1		
PD (11)	4.978 (2.208-11.224)	3.160 (1.424-7.017)		
**Change rate of PDGF-BB**			0.013*	0.001**
SR (37)	1	1		
RSR (31)	2.234 (1.181-4.223)	2.987 (1.539-5.797)		

SD, stable disease; PD, progressive disease; CR, complete response; PR, partial response; RSR, raised and slightly reduced; SR, significantly reduced. *, P < 0.05; **, P < 0.01; ***, P < 0.001.

### High PDGF-BB expression occurs in clinical cancer specimens and ESCC cell lines

**Figure 2:**
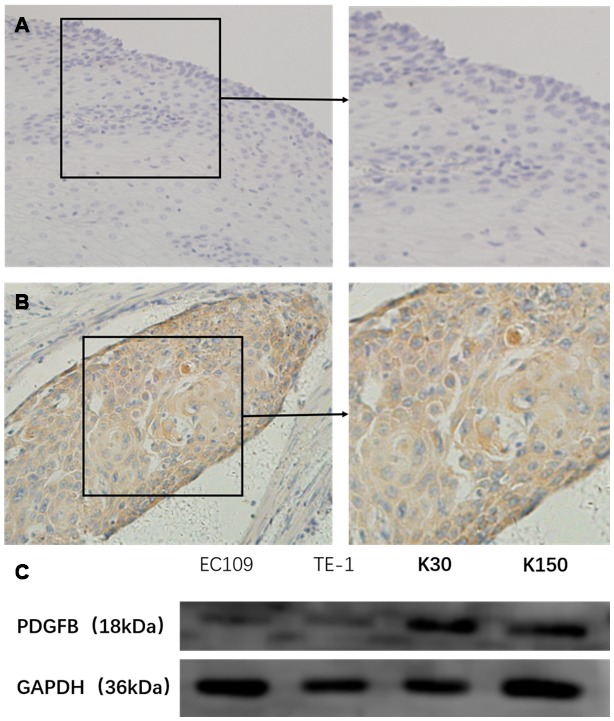
PDGF-BB expression in esophageal squamous cell carcinoma tissues and cell-lines was studied in a total of 41 cancer tissues (62.1%) of all 66 cases, and these cases had high expression (score: < 2) in PDGF-BB (**A**) while the expression was low in all 4 normal adjacent operative tissues (**B**) by immunohistochemistry. In addition, Western blotting showed that the levels of PDGFB were positively expressed in the 4 esophageal squamous cell carcinoma cell-lines (**C**).

We first detected PDGF-BB expression in 28 specimens of post-operative tumor tissues and four samples of normal adjacent tissue as the control. Among them, seventeen cases (60.7%) had strong expression (score: > 2, Figure 2A). The expression in four samples of normal adjacent tissue were all negative (Figure 2B). For the tissues sampled under endoscopy before radiotherapy, IHC revealed that 24 of the 38 patients (63.1%) had strong expression (score: > 2) of PDGF-BB. Taken together, the expression of PDGF-BB was upregulated in 41collected cancer tissues (62.1%) of all 66 ESCC samples. And then, we discovered that all four indicated ESCC cell-lines expressed postive levels of PDGFB by Western immunoblotting analysis (Figure 2C).

### Depletion of PDGFB inhibits ESCC cells growth and induces apoptosis

**Figure 3:**
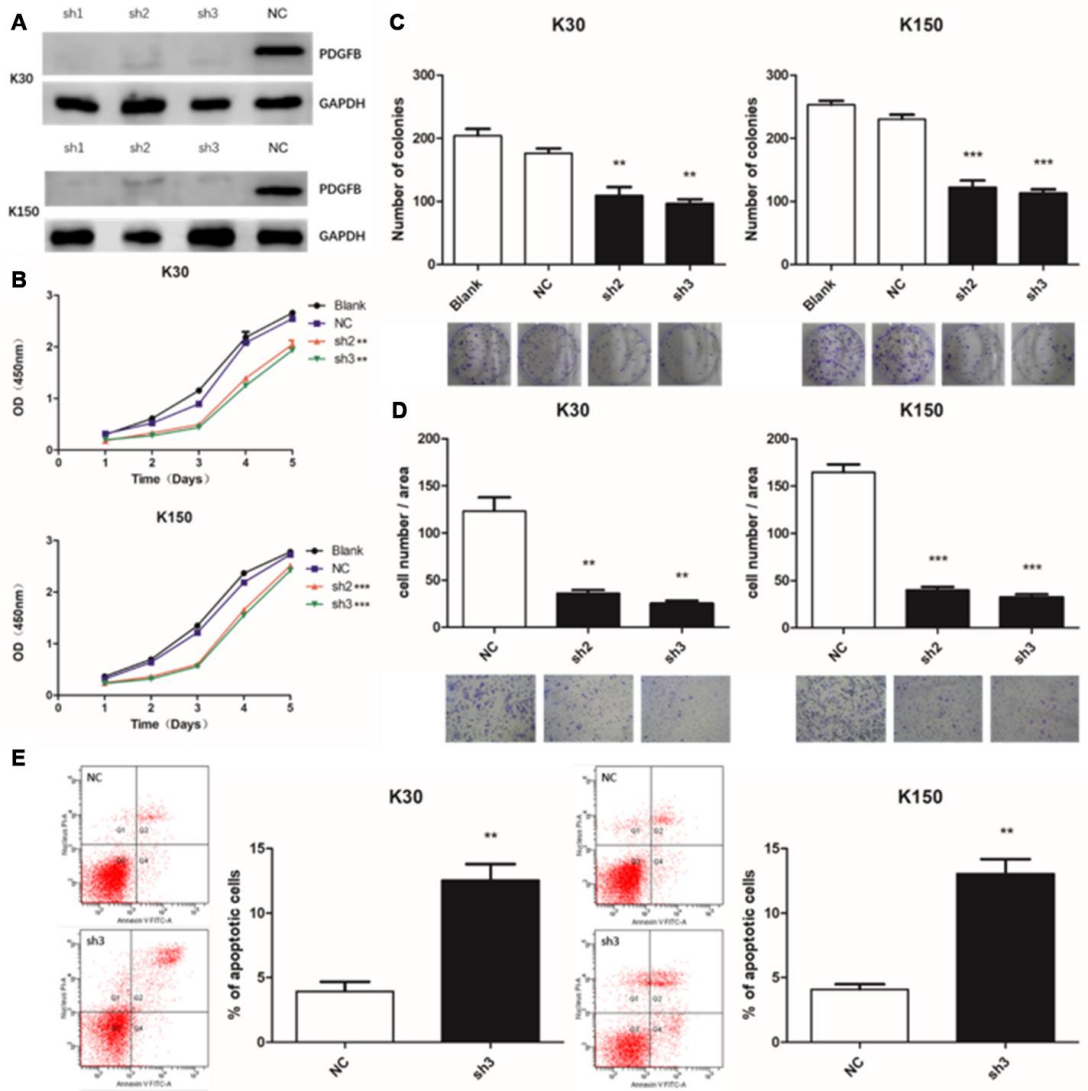
**Depletion of PDGFB inhibits esophageal squamous cell carcinoma cell growth and induces apoptosis.** Western blotting analysis showed that PDGFB expression was down-regulated after lentiviral transduction (**A**). Depletion of PDGFB resulted in a lower proliferation rate in KYSE30 and KYSE150 cells as determined by the Cell Counting Kit-8 (**B**) and colony formation assays (**C**). In addition, the transwell assay was applied to show that PDGFB depletion also reduced cell invasion (**D**). Depletion of PDGFB could also promote apoptosis of both cell-lines (**E**). **P < 0.01, ***P < 0.001.

Next, KYSE30 and KYSE150 cell-lines were chosen for further study because of their higher expression of PDGFB. Both cell-lines were effectively transduced by lentivirus carrying the PDGFB shRNA (sh1, sh2 and sh3) and the corresponding control scrambled shRNA (negative control). Knock-down efficiency was also confirmed by Western blotting and all of them achieved good effects (Figure 3A).

Here, we applied the CCK-8 and colony formation assays to assess cell proliferation. The results showed that depletion of PDGFB resulted in a lower proliferation rate in KYSE30 and KYSE150 cells (Figure 3B and 3C). Moreover, the transwell assay was applied to show that PDGFB depletion also reduced cell invasion in both ESCC cell-lines (Figure 3D). Next, we conducted studies with the Annexin V-APC/PI apoptosis assay and observed that PDGFB gene silencing promoted the apoptosis of both cell-lines (Figure 3E). Put together, these results suggest that depletion of PDGFB inhibits ESCC cell growth and induces apoptosis.

### Inhibition of PDGFB enhances the sensitivity of ESCC cells to IR and IR suppresses PDGF-BB-induced migration by blocking PI3K/AKT pathway

**Figure 4:**
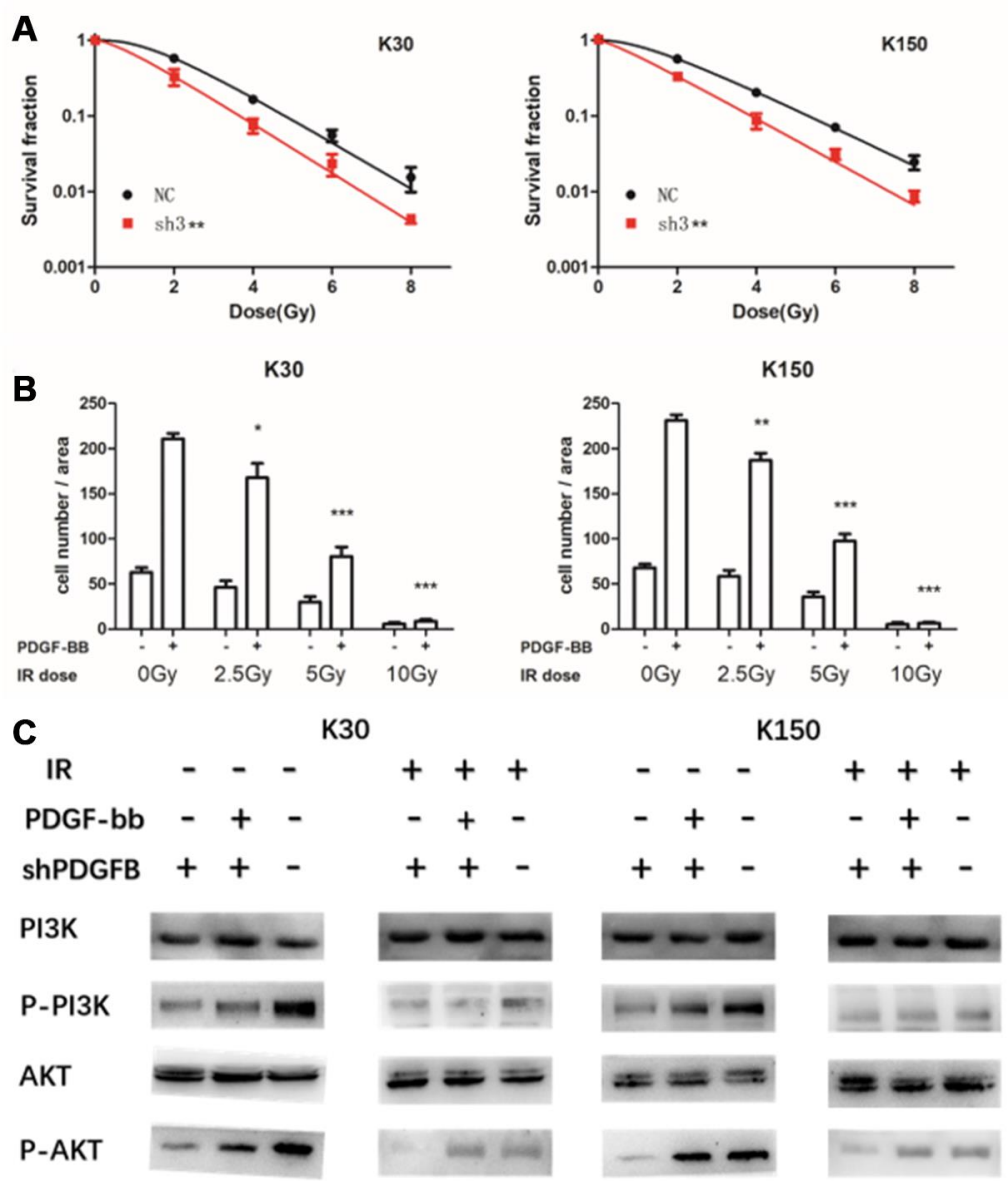
**Inhibition of PDGFB enhanced the sensitivity of esophageal squamous cell carcinoma cells to ionizing radiation (IR), a treatment modality that suppressed PDGF-BB-induced migration, and did so by blocking the PI3K/AKT pathway.** PDGFB-knockdown in cancer cells showed lower colony formation capacity than control cells after IR (**A**). In addition, results of the transwell assay suggested that PDGF-BB can promote KYSE30 and KYSE150 cell migration while IR can suppress it (**B**). Western blot analysis showed that the PI3K/AKT pathway was activated by PDGF-BB, and pretreatment with IR suppressed PDGF-BB-induced phosphorylation of PI3K and AKT in both cell-lines (**C**). *P < 0.05, **P < 0.01, ***P < 0.001.

We studied the effect of PDGFB on the sensitivity of KYSE30 and KYSE150 cells to radiotherapy. Under the action of irradiation (2, 4, 6, and 8 Gy), PDGFB-knockdown of ESCC cells showed a lowered capacity for colony formation than control cells after IR (Figure 4A). This result suggests that inhibition of PDGFB increases radio-sensitivity in ESCC cells. Moreover, we next examined the effect of PDGF-BB on migration in ESCC cells and whether IR could affect it. Cells were pretreated with increasing doses of IR (2.5, 5, and 10 Gy) and were then exposed to the specified concentration of PDGF-BB (50ng/ml). We detected that PDGF-BB could cause migration in ESCC cells. As depicted, PDGF-BB increased the number of migrated cells at the same dose. In addition, when exposed to the indicated increasing doses of IR, the number of migrated cells had clearly decreased (Figure 4B). These results strongly suggest that PDGF-BB promotes the cellular migration of both the KYSE30 and KYSE150 cell-lines, while IR can suppress it.

In addition, to further assess how IR affects the migration induced by PDGF-BB in EC cells, we added PDGF-BB (50ng/ml) in knocked-down ESCC cells after IR (8 Gy) and collected the cells. Western immunoblotting analysis was performed to verify that protein expression was correlated with the response to PDGF-BB and IR pretreatment (Figure 4C). It showed that the PI3K/AKT pathway was significantly activated by the addition of PDGF-BB in both the KYSE30 and KYSE150 cell-lines, and pretreatment with IR suppressed PDGF-BB-induced phosphorylation of PI3K and AKT in both cell-lines. Thus, we concluded that IR inhibits PDGF-BB-induced migration by suppressing the PI3K/AKT pathway in ESCC cells (Figure 4B).

## DISCUSSION

To our knowledge, this is the first study of the expression of PDGF-BB in radiotherapy treated EC. PDGF-BB was previously reported to be significantly associated with many human tumors including colorectal cancer [10, 11], pancreatic cancer [11–13], non-small cell lung cancer [14], renal cancer [15], ovarian cancer [16], liver carcer [17, 18] and even osteosarcoma [19]. Current thinking is that over-expression of the PDGF family probably leads to a poor prognosis by modulating both hematopoiesis and tumor angiogenesis [20]. However, there are currently few research studies that have explored the relevance of PDGF-BB expression in EC.

Previously, Matsumoto et al. showed that expression of PDGF-BB was positive in 31 of 53 EC cases (58.5%) by IHC [8], the result of which was similar to our work (41/66, 62.1%). Then another study indicated that serum PDGF-BB was significantly higher in EC patients than in controls (3.76 vs. 2.66 μg/l, p = 0.0001) [21]. Both articles suggested that the high expression in EC was positively correlated with tumor progression. But the researches were not deep enough. Radiotherapy as a major treatment for ESCC has not been mentioned. And study of mechanism is still lacking.

In our research, we tested the expression levels in serum both before and under radiotherapy from ESCCs for the first time. These studies were conducted in order to obtain dynamic changes in PDGF-BB expression. However, serum samples were submitted for testing in a total of three tests across different seasons, which caused wide variance in the expression values of PDGF-BB. To avoid this error, we created a variable named change rate and defined it as (expression value under treatment - expression value before treatment)/ expression value before treatment. This study assessed the value of PDGF-BB expression as a prognostic indicator in patients with ESCC that were treated by radiotherapy. Finally, we found that changes in the serological levels of PDGF-BB was closely associated with the overall prognosis. Regarding the choice of electing surgical treatment or not, the change rate in the serum indicated that patients with significantly reduced PDGF-BB had a much improved prognosis than the other group in terms of both PFS and OS, and the change rate was also closely associated with response following radiotherapy (p=0.042).

In addition, our findings demonstrated that tumor location was closely associated with OS (p = 0.021 in univariate analysis and p = 0.022 in multivariate analysis). Since treatments vary in different subsections of thoracic esophageal carcinoma, the relationship between survival and location of the tumor was seldom discussed. Results from a population-based study demonstrated that 1-year or 5-year adjusted survival of early EC did not differ by the anatomical location of the tumor [22]. However, we found that patients with upper esophageal cancer exhibited a longer mean PFS and OS. Maybe tumors that are located in the upper third are more suitable for radiotherapy than surgery as compared to tumors that are located in the middle and lower zones.

Besides location, concurrent chemotherapy might also represent a prognostic factor for PFS by multivariate analysis (P=0.033). Combined with chemotherapy, there was an increased PFS time as compared to radiotherapy alone. That conclusion is similar to the results of the 8501 trial [5]; however, only six patients failed to receive concurrent chemotherapy in this study. The CROSS trial and the 5010 trial indicated that the OS was significantly improved in the chemo-radiotherapy–surgery group than in the surgery alone group [23–25]. It is worth noting that although surgery still appears to be the standard therapeutic method for middle and lower EC patients, analysis of the surgical results that were combined with neoadjuvant radiotherapy versus radical radiotherapy did not make any significant difference. However, due to the limitation of the sample capacity of this study (only 18 surgically operated on patients), we cannot jump to conclusions too readily.

The PDGF family is identified as an autocrine stimulator for the growth and survival of human ESCC cell-lines [26]. Experimental studies on PDGF-BB in ESCC are extremely rare. Thus, we further assessed how PDGFB could affect the growth of ESCC cells. Using the KYSE30 and KYSE150 cell-lines, we found that depletion of PDGFB could reduce cell proliferation and invasion, and promoted cellular apoptosis. Inhibiting PDGFB promoted the sensitivity of ESCC cells to IR. Moreover, IR inhibited PDGF-BB-induced migration by blocking the crucial downstream pathway PI3K/AKT, a signaling mechanism that is related to the occurrence of many kinds of human tumors. The PI3K/AKT signaling pathway is involved in the regulation of a myriad of cellular functions including cell proliferation, differentiation, apoptosis and glucose transport. It also plays an important role in tumor radiotherapy resistance. Various growth factors and signaling complexes, such as PDGF-BB, can initiate the activation of PI3K/AKT. Therefore, we considered that PDGF-BB-induced migration was mediated by the PI3K/AKT pathway. Interestingly, IR disrupted this phenomenon, and did so by disturbing the activation of PI3K/AKT signaling.

To summarize, for the first time, we found that the expression of PDGF-BB provides a possible model for predicting the response and prognosis of ESCC radiotherapy. It can be used as a prognostic indicator in patients with ESCC that were treated by radiotherapy, which should facilitate the individualization of ESCC treatment. This biomarker may be useful for selecting the patients which are more sensitive to radiation thus guiding the treatment strategy. However, our research also had some limitations due the paucity of a sufficiently large case study size. Additional clinical research with quantities of cases that are markedly increased is warranted to fully assess its predictive power.

## MATERIALS AND METHODS

### Serum samples and enzyme-linked immunosorbent assay (ELISA)

First, we selected four radiotherapy-sensitive cases and four radiotherapy-resistant cases of ESCCs that were obtained from patients treated with radical radiotherapy that was performed in the Department of Radiotherapy. Blood samples were collected from eight participants before and after radiotherapy at a dose of 40 Gy/20 f. Within 6 hours, serum was separated by centrifugation at 3000 rpm, for 15 min, at 4°C. The serum was stored at -80°C until assayed using Human PDGF-BB ELISA kits provided by Neobioscience (Shenzhen, China) according to the manufacturer’s instructions strictly. And the absorbances were read at 450 nm with an Expert 96 microplate reader. We found that the expression of PDGF-BB was down-regulated respectively at 2.831 times (p = 0.034) after radiotherapy. We further expanded the sample content to verify the association of PDGF-BB with prognosis. In these studies, 68 cases were examined in the same way and clinical data were also collected.

The whole sample included 60 male and 8 female patients, with a median age of 60 years (range: 47-79 years). Among them, 18 patients underwent radical resections after radiotherapy with 40 Gy/20 f. The remaining 50 patients received radical radiotherapy at a total dose of 60 Gy/30 f. Concurrent chemotherapy was provided to 62 patients. All patients had received radiotherapy in our hospital from 2015 to 2017 and were fully informed about the study (provided signed and written consent).

### Evaluation and classification of patients by ELISA

The condition of the patients was assessed according to the system for staging primary tumor/regional lymph nodes/distant metastasis (TNM) as described in the 7^th^ Union for International Cancer Control (UICC) Cancer Staging Manual. Tumor development was examined by endoscopic ultrasonography, esophagography and spiral CT scan. Evaluation was classified according to the Response Evaluation Criteria in Solid Tumors (RECIST) 1.1.

Progression-free survival (PFS) was defined as survival from the date of starting treatment until the date of the first clinical evidence of disease progression or death. And overall survival (OS) was defined as survival from the date of starting treatment until date of death from EC or the treatment.

The change rate was defined as (the expression value under treatment – the expression value before treatment)/the expression value before treatment. Data was divided into two groups (raised and slightly reduced n = 31; and significantly reduced n = 37) due to change rates of PDGF-BB.

### Tissue specimens and Immunohistochemistry (IHC) evaluation

The expression of PDGF-BB in 28 non-neoadjuvant radiotherapy cases of cancer and four normal adjacent operative tissues were first tested by IHC. In addition, the other 38 treatment-naive cases of ESCC tissues were collected prior to radiotherapy by endoscopic bite detection. These tissues were stored at -80°C. Paraffin-embedded tissues were cut into 4 μm sections and rehydrated through a graded alcohol series, subjected to heating for antigen retrieval by EDTA antigen retrieval solution (ZSGB-BIO, Beijing, China), allowed to cool to room temperature, and then immersed in 3% hydrogen peroxide for 5–10 min, followed by rinsing in phosphate-buffered saline. The sections were incubated with normal goat serum to block non-specific binding, followed by incubation overnight with primary antibody that recognized PDGF-BB (host: rabbit; reactivity: human; Abcam, UK) at 4°C, and then rinsed with PBS. Subsequently, the sections were incubated with a secondary antibody (host: goat; reactivity: rabbit; ZSGB-BIO, Beijing, China) for 30 min at 37°C, washed with PBS, and then stained with diaminobenzidine. Finally, the sections were counterstained with hematoxylin, then dehydrated, and mounted.

The expression was assessed by three independent pathologists (Puchun Er, Dong Qian and Wencheng Zhang) who were blinded to clinical data. For evaluation of staining, a semiquantitative scoring criterion was used, in which both staining intensity and positive areas were recorded. A staining index (values 0–6) obtained as the intensity of positive staining (non-staining; 0; yellow, 1; claybank, 2; tawny, 3) and the proportion of immune-positive cells of interest (<10%,0; 10–39%, 1; 40–69%, 2; ≥70%, 3) were calculated. At last, cases were classified into two different groups: low-expression cases (score 0–2) and high-expression cases (score 3–6).

### Cell lines

The human ESCC cell-lines KYSE30, KYSE150, Ec109 and TE-1 were obtained from the Tianjin Key Laboratory. The cells were maintained in RPMI-1640 (Gibco Laboratories, USA), while Ec109 and TE-1 were maintained in DMEM (Gibco Laboratories, USA), which was supplemented with 10% fetal bovine serum (FBS) (Gibco Laboratories, USA) and 1% penicillin–streptomycin antibiotics (Gibco Laboratories, USA) at 37 °C in a 5% CO_2_ incubator.

### Lentivirus infection and generation of stable cell line

Lentivirus carrying PDGFB were obtained from Genechem Co., Ltd., Shanghai, China. The target sequences of PDGFB for constructing lentiviral short hairpin RNA (shRNA) were as follows: 5′- TTAAGAAACGAGCGAACAA -3′(shRNA1), 5′- TTAAGAAACGAGCGAACAA -3′(shRNA2) and 5′- AAGTGATGACGACTCTTAT -3′(shRNA3). In addition, 5′- TTCTCCGAACGTGTCACGT -3′ was chosen as the negative control. For transfection, cells were plated at a density of 1 x 10^5^ cells/well in six-well plates. After 20 hours of culture, 40 μL of HiTransG liquid and the corresponding virus suspension were added into each well according to the manufacturer’s recommendations. Transfected cells were cultured at 37°C with 5% CO_2_ for 24 hours before being selected in medium that was supplemented with 1.5 μg/mL puromycin (Solarbio, Beijing, China) for approximately two days. After transfection, cells were transferred to 10-cm cell culture dishes to generate and propagate stable cell-lines. Inhibition of PDGFB was identified by Western immunoblotting.

### Western blot assay and antibodies

Cells were lysed in lysis buffer. The BCA kit was used to determine protein concentrations. 10% SDS-PAGE gels and Western immunoblotting were done according to standard procedures. Proteins were detected with antibodies recognizing PDGFB (Abcam, UK), PI3K (Cell Signaling Technology, USA), phosphor-PI3K (Abcam, UK), AKT (Cell Signaling Technology, USA) and phosphor-AKT (Cell Signaling Technology, USA). GAPDH (Cell Signaling Technology, USA) was used as a protein loading control.

### Cell proliferation assay

Cell viability was measured with the use of the Cell Counting Kit-8 (CCK-8) (Beyotime Biotechnology, Shanghai, China) assay. Briefly, cell suspensions containing about 2500 cells were seeded into each well of a 96-well plate and cultured at 37°C with 5% CO2. Cell viability was determined following standard procedures. All experiments were done in triplicate.

### Invasion assay

The invasive ability of ESCC cells in vitro was evaluated by a transwell assay using Matrigel Invasion Chambers (Corning Costar Corp., Cambridge, MA). In this assay, 80000 cells in 200 μl FBS-free medium were added to the upper chamber of a Transwell and 10% FBS-containing medium was added to the lower chamber. After 16 h, the cells were fixed in 4% paraformaldehyde and stained by Giemsa (Solarbio, Beijing, China). The cells were then observed under a light microscope and the number of migrating cells was counted in five randomly chosen fields on the lower surface of the membrane. All data were obtained from at least three independent experiments.

### Annexin V-APC/PI flow cytometry apoptosis assay

Annexin V-APC and PI stains were used to determine the percentage of cells undergoing apoptosis. The apoptosis assay was conducted using the protocol supplied by the manufacturer (BD Biosciences, Bedford, MA). Each sample was then subjected to analyses by flow cytometry (BD FACSCanto II Flow Cytometer, BD Biosciences).

### Clonogenic survival assay

Cell survival following radiation exposure was defined as the ability of the cells to maintain clonogenic capacity and to subsequently form colonies. Cells were counted and seeded for colony formation in six-well plates at 200–3200 cells per well. After attaching to the culture flask, cells were exposed to the indicated doses (0-8Gy) of ionizing radiation (IR) using 6 MV X-rays from a linear accelerator. Cells were incubated at 37°C of 12 days after irradiation. Colonies were stained with crystal violet and manually counted. Colonies consisting of 50 cells or more were scored. All experiments were done in triplicate.

### Migration assay

Cell migration was assessed using a Boyden chamber (8 μm pores, Transwell®; Corning Costar Corp., Cambridge, MA). Cells (80000 per well) were exposed to the indicated doses (0-10Gy) of IR using 6 MV X-rays from a linear accelerator and then incubated for 20 h. These cells were then added to the upper chamber of a Transwell containing 50 ng/ml recombinant human PDGF-BB (R&D Systems, WA, USA) and 10% FBS-containing medium was added to the lower chamber. After 24 h, the cells were fixed by the remaining steps in the invasion assay.

### Statistical analysis

Clinical data and the expression level assay data were calculated with the IBM SPSS Statistics software 24. Survival status was calculated empirically according to the Kaplan-Meier method and was measured from the day of radiotherapy. Association of the change rate of PDGF-BB with clinical variables was analyzed by the Chi-square test. Univariate and multivariate analysis of prognostic factors was performed basing on the Cox proportional hazards model. A probability level of P<0.05 indicated statistical significance.
